# An Outbreak of Coxsackievirus A6 Infection in Adults of a Collective Unit, China, 2019

**DOI:** 10.1155/2022/6607294

**Published:** 2022-08-27

**Authors:** Yumeng Gao, Guangyuan Ma, Yong Xiao, Qun Cai, Yujun Chen, Ping Shi, Kewei Wang, Yuan Shen, Chao Shi

**Affiliations:** ^1^Department of Acute Infectious Disease Prevention and Control, Wuxi Center for Disease Control and Prevention, Wuxi, Jiangsu, China; ^2^Microbiological Laboratory, Wuxi Center for Disease Control and Prevention, Wuxi, Jiangsu, China; ^3^Department of Acute Infectious Disease Prevention and Control, Hui Shan Center for Disease Control and Prevention, Hui Shan, Wuxi, Jiangsu, China; ^4^Department of Nosocomial Infection, Affiliated Hospital of Jiangnan University, Liangxi, Wuxi, Jiangsu, China; ^5^Department of Acute Infectious Disease Prevention and Control, Wuxi Center for Disease Control and Prevention, The Affiliated Wuxi Center for Disease Control and Prevention of Nanjing Medical University, Wuxi, Jiangsu, China

## Abstract

Outbreaks/epidemics caused by coxsackievirus A6 (CVA6) have been reported continuously since 2008. However, outbreaks of ocular conjunctival hemorrhage caused by CVA6 in adults in a collective unit have not been reported. *Methods.* The epidemiological investigations were carried out according to the monitoring program, and the clinical data were collected from the treated hospitals. The nasopharyngeal swab specimens were collected to extract the total nucleic acid (DNA/RNA). The pathogen was determined using nucleic acid detection kits for 22 respiratory pathogens. The VP1 gene of this pathogen was amplified and sequenced. Sequence alignment and analysis were performed using BioEdit 7.0. The gene phylogenetic tree was constructed with MEGA4.0. *Results.* The factory emerged patients in succession from February 14 and reached the peak on the 18th. A total of 19 workers had symptoms in this factory up to March 31, 2019, giving an attack rate of 8.26%. The main symptoms were rash, ocular conjunctival hemorrhage, fever, and sore throat. The laboratory results showed that coxsackievirus A6 was the main pathogen causing this outbreak. The risk of taking a bath in the bathroom was 7.37 times higher than that of not taking a bath (95% confidence interval (CI): 1.67–32.79). *Conclusion.* This manuscript further enriched the infection-related information of CVA6, which was helpful to better identify and deal with the epidemic in the future.

## 1. Introduction

Human enteroviruses are RNA viruses in the genus *Enterovirus* of the *Picornaviridae* family, which can cause a wide range of clinical manifestations [1]. They are classified into four species (A-D). Coxsackievirus A6 (CVA6) belongs to species A [2]. Unlike enterovirus 71 (EV71) and coxsackievirus A16 (CVA16), CVA6 infection can cause many atypical clinical manifestations and even lead to severe central nervous system disorders [3, 4]. Currently, CVA6-related vaccines are not yet available in China. The public health threat it caused cannot be ignored.

Since 2008, epidemic caused by CVA6 infection has been reported continuously [5–7]. In China, outbreaks/epidemics caused by this pathogen increased after 2013, and even replaced EV71 and CVA16 in many areas, becoming the dominant strain causing hand, foot, and mouth disease (HFMD) [8, 9]. However, outbreaks in adults with ocular conjunctival hemorrhage caused by CVA6 infection in a collective unit have not been reported. In February 2019, an outbreak caused by CVA6 infection in adults occurred at a pharmaceutical factory in Wuxi, China. In this study, we aimed to elaborate on epidemiological characteristics, clinical manifestations, risk factors, and laboratory testing about this outbreak.

## 2. Materials and Methods

### 2.1. Sources of Data

The daily surveillance data, epidemiological investigation information, and outbreak data of HFMD were collected according to“The Monitoring Work Plan for HFMD in Wuxi,” and from “Public Health Emergency Reporting Management Information System,” respectively. Meanwhile, we collected the clinical information of cases from the treated hospitals.

### 2.2. Case Definition

HFMD outbreak: Within a week, 10 or more HFMD cases occurred in the same nursery or school or other collective units, or 5 or more HFMD cases occurred in the same natural village/neighborhood committee.

### 2.3. Specimen Collection

The nasopharyngeal swab specimens were collected from 12 of the patients and placed in sampling tubes containing 3 ml of virus sampling solution immediately on the spot. Then, the specimens were immediately sent to the laboratory at 4°C for respiratory tract pathogen nucleic acid detection.

### 2.4. Extraction and Detection of Viral Nucleic Acid

The total nucleic acid (DNA/RNA) was extracted using a Roche MagNA Pure LC2.0 fully automated nucleic acid extractor (Roche Applied Science, IN, USA) with the Roche MagNA Pure LC total nucleic acid isolation kits after instructions. The extracts were dissolved in 100 *μ*l eluate and immediately stored in a −70°C refrigerator.

The pathogen was determined using nucleic acid detection kits for 22 respiratory pathogens (Shanghai GeneoDX Biotech Co. Ltd., Shanghai, China). The detection was conducted, and results were interpreted according to the kit's instructions.

### 2.5. Amplification and Sequencing of Enterovirus' VP1 Gene

The VP1 gene primers (F: 5′-AYCYTTGTRCGCCTGTTTT-3′, R: 5′- CCCAAAGTGTCGGTTCCGC-3′) were synthesized by Sangon Biotech (Shanghai) Co., Ltd. Detection was performed using the QIAGEN One-Step RT-PCR Kit (cat. no. 210212, Germany). The 25*μ*l reaction system consisted of 5 ×buffer 5 *μ*l, 10 mM dNTP mixture 1 *μ*l, enzyme mix 1 *μ*l, upstream primer (20 mM) 0.5 *μ*l, downstream primer (20 mM) 0.5 *μ*l, H_2_O treated by DEPC 13 *μ*l, and RNA 4 *μ*l. The PCR conditions were 45°C for 30 min, 95°C for 15 min, 30 s at 94°C, 30 s at 56°C, and 72°C for 1 min (total 40 cycles), with a final extension at 72°C for 10 min. The amplified products were sent to Shanghai Majorbio Bio-Pharm Technology Co., Ltd., for sequencing.

### 2.6. Phylogenetic Analysis

The 6 samples positive for CVA6 were aligned with reference sequences using the Clustal W program implemented in BioEdit 7.0. The reference sequences that represented all known CVA6 subgenotypes were obtained from the GenBank database. Neighbor-joining (NJ) method was employed to construct phylogenetic tree with bootstrap method 1000 as a parameter in MEGA 4.0.

### 2.7. Data Analysis

We performed descriptive epidemiologic analyses based on Mill's canon to generate hypotheses on risk factors for epidemic situation spread. Categorical variables were presented as numbers and percentages. Fisher's exact test was used to compare attack rates among different departments. The chi-squared test was used to compare the difference between the incidence of bathing in the bathroom of the factory and not taking bath in the bathroom. Risk factor analysis was performed using odds ratio (OR). Analyses were performed with SPSS version 11.0 (SPSS, Chicago, IL, USA). All testing was two-sided, and *p* < 0.05 was considered statistically significant.

## 3. Results

### 3.1. Epidemiological Characteristics and Clinical Features

**Figure 1:**
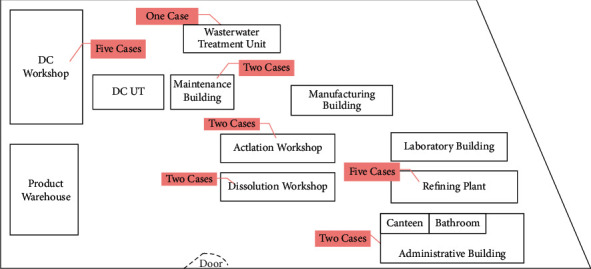
Workshop distribution plan of the factory.

The outbreak occurred in a pharmaceutical factory of Wuxi, China. The factory had 238 employees in 13 departments, of which 7 departments were involved in this outbreak (Figure 1). The index case A was in the DC workshop of the production department. On February 14, 2019, case A consciously developed fever, but did not measure body temperature. The next day around 11pm, the patient found a lot of rashes (no itching) on her arms and torso, accompanied by sore throat. On the third morning, the patient developed symptoms of dry mouth, ocular conjunctival hemorrhage, and the rashes spread over her face and body. Then, she went to the hospital for treatment. The WBC count was 2.92^*∗*^10^9^/L. The doctor gave her antiviral treatment. The symptoms of ocular conjunctival hemorrhage disappeared on the fourth day. The rashes subsided, and the body was itchy next day. According to the patient's self-report, she and her family traveled to Suzhou, Shanghai, and Zhoushan, respectively, from February 5 to February 7, with one day in each place. Zhoushan is a coastal city with abundance of seafood. The patient ate a large amount of seafood at street stands during the tour in Zhoushan on February 7, including oysters, scallops, fish, shrimp, and so on. The food ingested by the patient in Shanghai and Suzhou was healthy, without eating at street stands, no history of aquatic products except fish and freshwater shrimp, no raw, and cold food. During the travel, the patient felt a little tired and weak. The patient had not been exposed to similar cases, and there were no children with HFMD in his family. She returned to work on the 11th until 15th.

**Figure 2:**
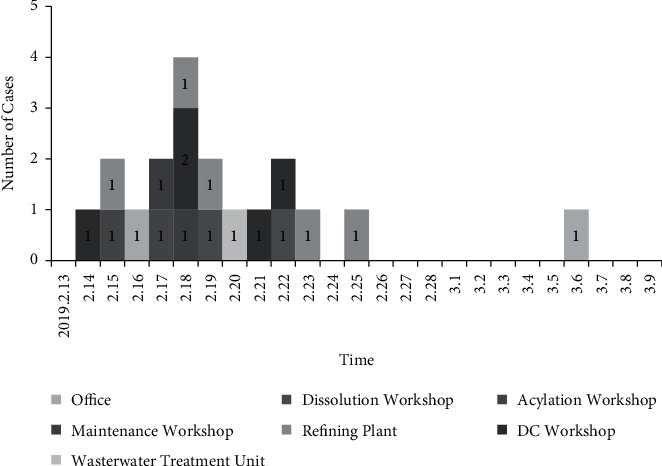
Epidemic curve of 19 cases involved in this outbreak.

The factory emerged patients with rash, fever, and ocular conjunctival hemorrhages as the main symptoms in succession from February 15. It spreads to other workshops and reached the peak on the 18th. After taking the control measures such as disinfection, window opening, and ventilation, home isolation on the 19th, the number of cases decreased, but still had cases. In order to better control the outbreak, the factory was temporarily closed on 23rd for 10 days. Eventually, the outbreak ended on March 6 (Figure 2).

The highest percentage for spatial distribution of cases was in the DC workshop, where the first case was located, and refining plant (26.32%, respectively), followed by dissolution workshop, maintenance workshop, acylation workshop, and office (10.53%, respectively) (Figure 1). According to the epidemiological investigations, all employees involved in the production of the products left work after taking a bath in the factory's bathroom. Of the patients who developed the disease, 89.47% (17/19) used the bathroom. All patients had no history of exposure to similar cases outside the factory.

**Figure 3:**
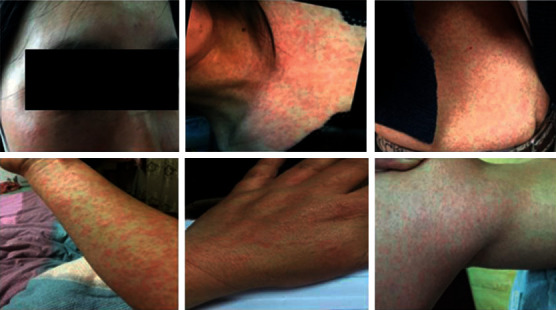
Typical clinical symptoms of the patients.

A total of 19 workers had symptoms up to March 31, 2019, giving an attack rate of 8.26%. These patients with a male to female ratio of 1.11 to 1 were between 22 and 42 years old. The main symptoms were rash (19 cases, 100.00%), ocular conjunctival hemorrhage (19 cases, 100.00%), fever (total 11 cases, 57.89%: 4 cases below 38.5°C, 5 cases of 38.5–40.5°C, and 2 cases of conscious fever, accounting for 21.05%, 26.32%, and 10.53%, respectively) and sore throat (6 cases, 31.58%). Except for one patient whose main symptom was ocular conjunctival hemorrhage, all other patients had systemic rashes, and some patients (7 cases, 36.84%) showed symptoms of fatigue and limb joint pain in the course of disease (Figure 3, Table 1). After symptomatic treatment by doctors (mainly antipyretic, anti-allergic, and antivirals), the patients experienced symptoms for an average of 8 days. Two patients (10.53%) felt itchy skin at the time of the eruption, and one patient presented with the debridement of the corners of the mouth. Five patients were not routinely examined for blood. Of the remaining 14 patients, except for 3 patients with low WBC counts, the rest was normal. Details of the cases are shown in Table 1.

### 3.2. Analysis of Risk Factors

Analysis of the attack rates in different departments showed that there was no statistical difference of the attack rates among various departments (*p* > 0.05) (Table 2).

We analyzed bathing in the bathroom as a risk factor for disease through the epidemiological investigations. The result showed that the risk of taking a bath in the bathroom was 7.37 times higher than that of not taking a bath (95% CI: 1.67–32.79) (Table 3).

### 3.3. Laboratory Results

**Figure 4:**
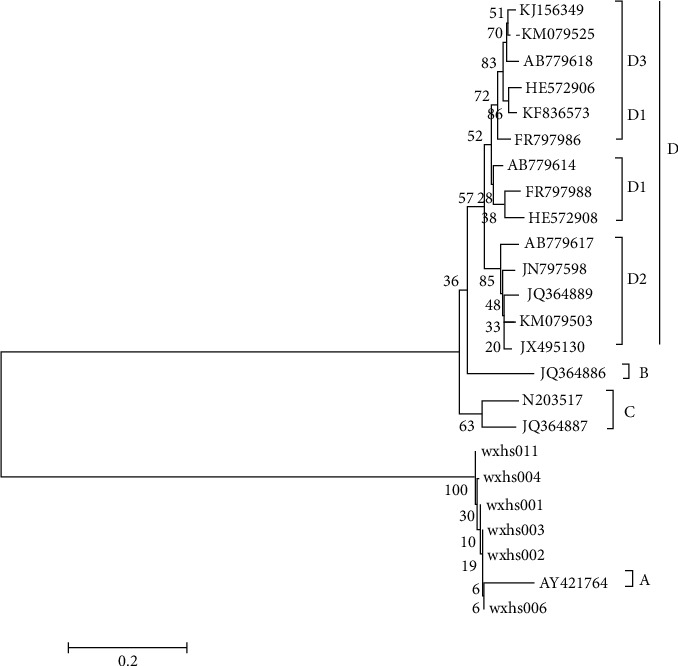
The phylogenetic tree of the six positive samples' VP1 genes.

Six of 12 nasopharyngeal swabs were positive for enterovirus nucleic acid. Subsequently, the VP1 genes of the six samples were amplified, sequenced, and identified by PCR. The sequencing results were analyzed by BLAST. The six samples became a cluster through comparison and analysis of the phylogenetic tree, which was the same branch as the original strain in the United States in 1949 (AY421764/USA1949). The homology was 91.5% (Figure 4). We concluded that CVA6 was the primary pathogen causing this outbreak.

## 4. Discussion

The epidemic caused by CVA6 gradually increased after 2008 [10–12]. Outbreaks in children/minors caused by CVA6 also occurred frequently [13–15]. However, the outbreak of CVA6-induced ocular conjunctival hemorrhage in adults in a collective unit was reported for the first time. According to the pathogenic surveillance results in Wuxi, EV71 (51.40%) and CVA16 (32.00%) were the main pathogens from 2013 to 2017. With the successive application of EV71 vaccine in 2017, CVA16 (49.69%) and CVA6 (36.21%) dominated in 2018–2019. Since 2018, HFMD outbreaks caused by enteroviruses other than EV71 and CVA16 have been typed in Wuxi and a total of 6 outbreaks (19.35%) caused by CVA6 were reported in “Public Health Emergency Reporting Management Information System” in 2018-2019. In this study, CVA6 was identified as the main pathogen, which was in line with the epidemic background of Wuxi in 2019.

According to the epidemiological investigation data, we inferred that the index case A might be infected with CVA6 during her travel, and then, she returned to work in the factory, causing further transmission. This assumption was based on the following findings: firstly, case A ate a large amount of seafood during the tour in Zhoushan on the 7th, including shellfish such as oysters, scallops, fish, and shrimp. Seafood was not handled cleanly or not cooked properly, which was prone to infection after use [16, 17]. Secondly, case A traveled from February 5 to 7. On February 14, she developed symptoms. The time was consistent with the incubation period of coxsackievirus A (generally 4–7 days) [18]. Thirdly, case A had a tight travel schedule and felt fatigue during the travel, which might easily reduce the immunity and increase the risk of infection. Fourth, except for case A, none of the other cases had travel history, special dining history, or contact history with people with similar symptoms (including contact with children with hand, foot, and mouth disease) outside the factory.

The results of analytical research suggested that bathing in the bathroom of the factory was a risk factor for the spread (OR: 7.37, 95% CI: 1.67, 32.79). The employees involved in the production of the products all left work after taking a bath in the factory's bathroom. Of the patients who developed the disease, 89.47% (17/19) used the bathroom. CVA6 is mainly transmitted through contact with respiratory secretions, scabs, vesicle fluid, and feces of patients, as well as contact with pollutants [19]. The bathroom was narrow, wet, and had no windows. There was only a small door and the ventilation was poor, which was conducive to spread of the coxsackievirus.

As previously reported, CVA6 was mainly associated with outbreaks of HFMD, and clinical symptoms mainly included fever, skin rash, desquamation, and onychomadesis [15, 20]. The Chinese study in 2015 showed that the proportion of fever in CVA6 group (78.69%) was higher than that in other groups [21]. A case-control study in Tianjin, China, also showed that children with HFMD caused by CVA6 infection were more likely to develop fever (OR: 3.391, 95% CI: 2.493, 4.612) and rash on their limbs (OR: 2.568, 95% CI:1.742, 3.786) [22]. The same was found in this study. The range of skin rash caused by the virus was wide and often brought desquamation and/or onychomadesis. A prospective study from April 2014 to March 2015 in France showed that rashes caused by CVA6 could spread to the limbs and face [23]. In 2011, Spain reported that children under the four years of age developed papulovesicular rash on the palms, soles, buttocks, and mouth (not extend to the rest of the face) [24]. The outbreak caused by CVA6 in kindergarten in Beijing, and children had skin rash (100.0%), fever (84.3%), desquamation (68.6%), onychomadesis (43.1%), and even 3.9% of the children who lost all their fingernails [15]. An outbreak of HFMD caused by CVA6 in Basic Military Trainees in Texas in 2015 showed that 11% and 96% of patients had prodromal symptoms of fever and malaise, respectively, and these symptoms were typically followed by erosive stomatitis and a rash that began on the palms and soles [25]. Symptoms of fever, skin rash, desquamation, and onychomadesis caused by CVA6 infection have also been reported in adult sporadic cases [26–28]. Previous studies have shown that enteroviruses such as coxsackievirus A24 and enterovirus 70 can cause acute hemorrhagic conjunctivitis [29, 30]. All the infected patients involved in this outbreak showed ocular conjunctival hemorrhage, while the main pathogen was CVA6, and coxsackievirus A24 and enterovirus 70 were not detected, which further enriched the existing studies. In this study, all patients were adults. Except for one patient whose skin rash did not appear in the whole body, the rest of the patients were visible in the whole body (including the face). When the rash subsided, except for 2 patients who felt itchy skin and 1 patient who showed skin peeling at the corner of the mouth, there were no other symptoms. All patients did not appear nail matrixes or onychomadesis during the follow-up. These were different from the previously reported clinical symptoms induced by CVA6.

There were several limitations. First of all, we could not be absolutely sure of the source of this outbreak, and we could only make inferences based on the available data. Second, we were not able to successfully collect environmental samples from the bathroom because the factory had disinfected the bathroom before informing us. Third, in dealing with this outbreak, we were not considering asymptomatic and carriers of the virus. Although the other workers in the factory and the family members of the patients had no clinical manifestations, we did not collect their specimens and could not know their infection status.

## 5. Conclusions

In this outbreak of adults, CVA6 was the main pathogen. The clinical symptoms of patients were different from those previously reported in children or sporadic cases in adults infected with CVA6. This manuscript further enriched the infection-related information of CVA6, which was helpful to better identify and deal with the epidemic in the future.

## Figures and Tables

**Table 1:** Detailed information on this outbreak including 19 cases.

Clinical symptoms^*∗*^ and duration of symptoms																			
Case number	Age (year)	Sex	Department	Use the bathroom	Onset date	Fever and maximum temperature (℃)		Rash and site of occurrence		Itchy skin	Ocular conjunctival hemorrhage	Vomiting and diarrhea	Cough	Headache	Sore throat	Fatigue and limb joint pain	Duration of symptoms (days)	Routine blood test	Whether sampling and positive for enterovirus
wxhs001#	29	Female	DC Workshop	Yes	2019/2/14	1	Conscious fever	1	Systemic rashes	1	1	—	—	—	1	—	5	Low white blood cell counts	Yes, Yes

wxhs002	38	Male	Acylation Workshop	Yes	2019/2/15	1	38.5	1	Systemic rashes	—	1	—	—	—	—	—	12	Low white blood cell counts	Yes, Yes

wxhs003	40	Male	Refining Plant	Yes	2019/2/15	1	＜38.5	1	Systemic rashes	—	1	—	—	—	—	1	7	Normal	Yes, Yes

wxhs004	38	Female	HR Office	No	2019/2/16	1	＜38.5	1	Systemic rashes	—	1	1	—	1	—	1	10	Normal	Yes, Yes

wxhs005	32	Male	Acylation Workshop	Yes	2019/2/17	1	38.5	1	Systemic rashes	—	1	—	1	—	1	—	8	Low white blood cell counts	Yes, No

wxhs006	22	Male	Maintenance Department	Yes	2019/2/17	1	39	1	Systemic rashes	—	1	—	1	1	1	—	6	Normal	Yes, Yes

wxhs007	39	Female	Refining Plant	Yes	2019/2/18	—	—	1	Systemic rashes	—	1	—	—	—	—	1	21	Normal	No, No

wxhs008	36	Male	Maintenance Department	Yes	2019/2/18	1	Conscious fever	1	A little rash on the trunk	—	1	—	1	—	—	—	5	Normal	Yes, No

wxhs009	31	Female	DC Workshop	Yes	2019/2/18	—	—	1	Systemic rashes	—	1	—	—	—	1	1	5	Normal	Yes, No

wxhs010	35	Female	DC Workshop	Yes	2019/2/18	—	—	1	Systemic rashes	—	1	—	—	—	1	—	14	Normal	Yes, No

wxhs011	42	Male	Dissolution Workshop	Yes	2019/2/19	—	—	1	Systemic rashes	—	1	—	—	—	—	—	3	Normal	Yes, Yes

wxhs012	35	Female	Refining Plant	Yes	2019/2/19	—	—	1	Systemic rashes	1	1	—	—	—	—	1	10	Normal	No, No

wxhs013	33	Female	Wasterwater Treatment Unit	Yes	2019/2/20	1	40.5	1	Systemic rashes	—	1	—	—	—	—	1	7	Normal	Yes, No

wxhs014	39	Female	DC Workshop	Yes	2019/2/21	—	—	1	Systemic rashes	—	1	—	—	—	1	—	10	Normal	No, No

wxhs015	35	Male	Dissolution Workshop	Yes	2019/2/22	—	—	1	Systemic rashes	—	1	—	—	—	—	—	7	Not done	No, No

wxhs016	39	Male	DC Workshop	Yes	2019/2/22	1	<38.5	1	Systemic rashes	—	1	—	—	—	—	—	8	Not done	No, No

wxhs017	42	Male	Refining Plant	Yes	2019/2/23	—	—	1	Systemic rashes	—	1	—	—	—	—	1	9	Not done	No, No

wxhs018	37	Male	Refining Plant	Yes	2019/2/25	1	<38.5	1	Systemic rashes	—	1	—	—	—	—	—	7	Not done	No, No

wxhs019	40	Female	HR Office	No	2019/3/6	1	38.8	1	Systemic rashes	—	1	—	—	—	—	—	9	Not done	Yes, No

^*∗*^1: have symptom, #index case.

**Table 2:** Comparison of attack rates in different departments.

Department	Total number	Number of cases	Attack rate (%)	*P*
DC workshop	39	5	12.82	0.57
Refining plant	29	5	17.24	
Acylation workshop	23	2	8.70	
Dissolution workshop	17	2	11.76	
Maintenance department	24	2	8.33	
HR office	5	2	40.00	
Wasterwater treatment unit	6	1	16.67	

**Table 3:** Analysis of the relationship between using bathroom and disease.

Using bathroom	Disease		*χ*2	*P*	Or (95% CI)
	Case	Control			
Yes	17	113	9.15	0.0025	7.37 (1.67, 32.79)
No	2	98			

## Data Availability

The datasets generated and analyzed during the current study are not publicly available due to the patients' privacy but are available from the corresponding authors on reasonable request after filtering out sensitive personal information of patients.

## Abbreviations

CVA6: Coxsackievirus A6
EV71: Enterovirus 71
CVA16: Coxsackievirus A16
CVA10: Coxsackievirus A10
HFMD: Hand, foot, and mouth disease
OR: Odds ratio
CI: Confidence interval
MGPs: Magnetic glass particles
